# Editor's Note

**DOI:** 10.1289/ehp.121-a177

**Published:** 2013-06-01

*EHP*’s News section has seen several changes since it began in 1993; sections have been added and deleted as times have changed. Our adoption of an online-only format has provided a natural opportunity to once again reassess how the News section can best serve readers. Today we are pleased to announce some changes starting with this issue.

First, we are enhancing how we communicate about *EHP* research by significantly expanding our Science Selections articles, which summarize research content published in the concurrent issue. We’ll be publishing longer Science Selections that include outside comment and explore the translational element of the research, making it more accessible and relevant for a wider audience. Second, we will no longer publish our short Forum or Beat articles. We will use Facebook and Twitter to alert readers to the current events we have traditionally covered in those formats. Our in-depth feature articles will continue unchanged.

Let us know what you think of the new, improved Science Selections. And be sure to check us out on Facebook and Twitter.

